# Development of Colonic Perforation during Calcium Polystyrene Sulfonate Administration: A Case Report

**DOI:** 10.1155/2013/102614

**Published:** 2013-12-10

**Authors:** Nobuhiro Takeuchi, Yusuke Nomura, Testuo Meda, Masato Iida, Akihito Ohtsuka, Kazuyoshi Naba

**Affiliations:** ^1^Department of Internal Medicine, Kawasaki Hospital, 3-3-1 Higashiyama-cho, Kobe, Hyogo 652-0042, Japan; ^2^Department of Laboratory Medicine, Kawasaki Hospital, 3-3-1 Higashiyama-cho, Kobe, Hyogo 652-0042, Japan

## Abstract

A 90-year-old female complaining of severe upper abdominal pain was transferred to our institution. The patient had been prescribed with calcium polystyrene sulfonate (CPS) for the treatment of hyperkalemia following myeloperoxidase-antineutrophil cytoplasmic antibody (MPO-ANCA) associated glomerulonephritis. Physical examination revealed diffuse tenderness over the abdomen, with signs of peritoneal irritation. Abdominal computed tomography (CT) revealed the retention of ascites, free air in the abdominal cavity, and the retention of hard stools in the left-sided colon. The diagnosis of intestinal perforation was immediately confirmed; thereafter, the patient underwent emergency surgical treatment. Surgical findings revealed a perforated site in the descending colon surrounded with hard stools. Histopathology of the perforated colon revealed crystalline materials, suggestive of association with CPS. CPS is a cation-exchange resin used to treat hyperkalemia; the major adverse effect in patients receiving CPS is constipation. When CPS is administered to patients with frequent constipation or the elderly, the risk of intestinal perforation should be considered.

## 1. Introduction

Calcium polystyrene sulfonate (CPS) is a cation-exchange resin widely used in the treatment of hyperkalemia associated with acute or chronic renal failure; however, the major side effect of CPS is constipation. Here we describe the case of a 90-year-old female who presented with colonic perforation during CPS administration.

## 2. Case Presentation

**Figure 1:**
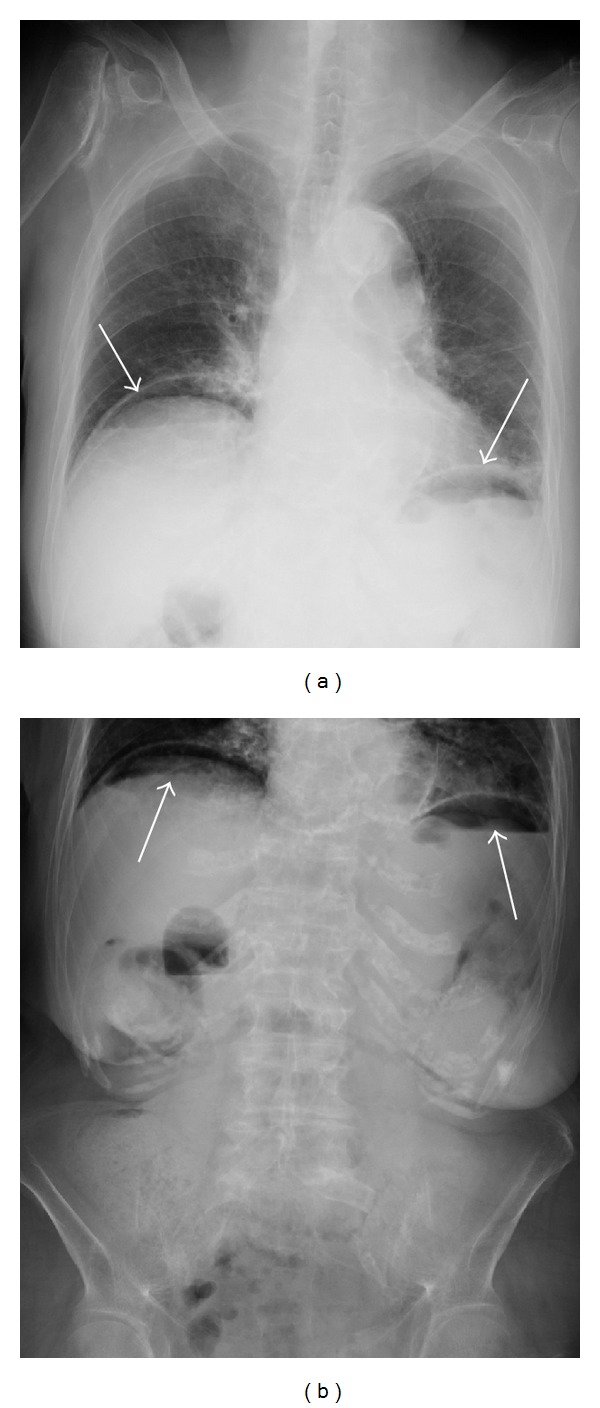
Chest (a) and abdominal (b) radiography revealed free air under bilateral diaphragms.

**Figure 2:**
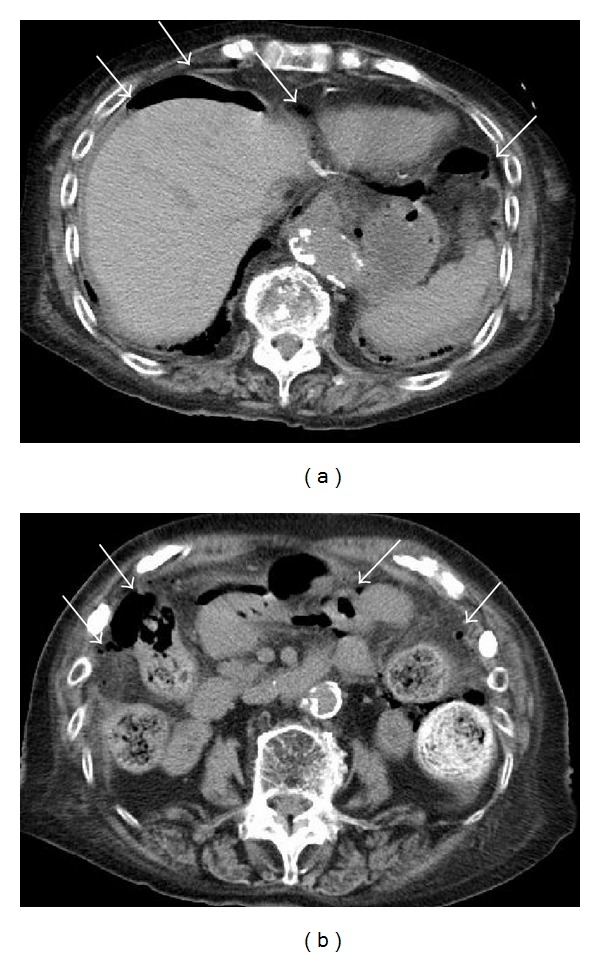
Abdominal computed tomography revealed the presence of ascites, free air in the abdominal cavity, and hard stools in the left-sided colon ((a), (b)).

**Figure 3:**
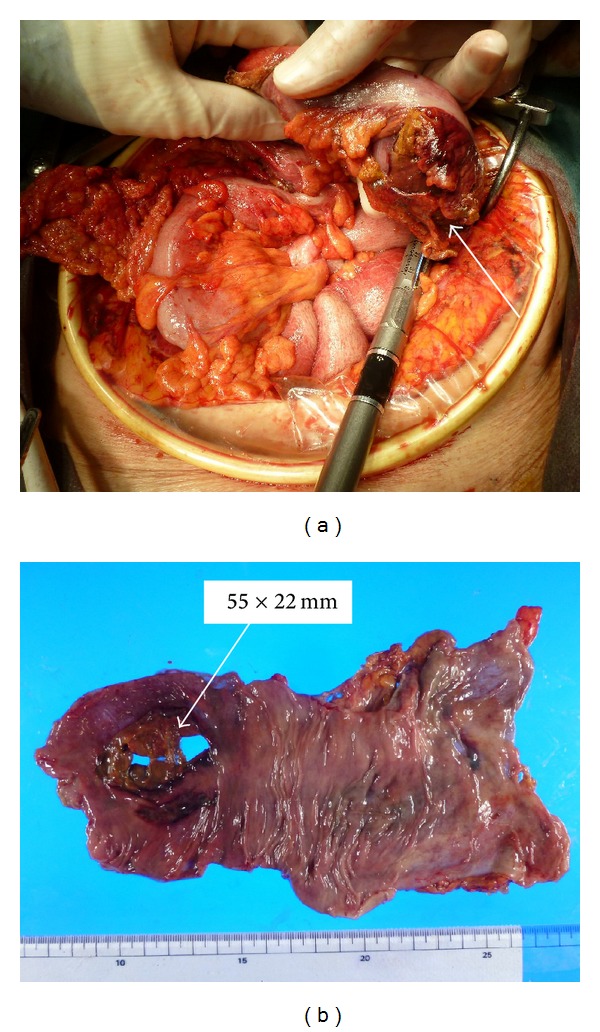
Surgical findings revealed a perforated site in the descending colon surrounded with hard stools ((a), (b)).

**Figure 4:**
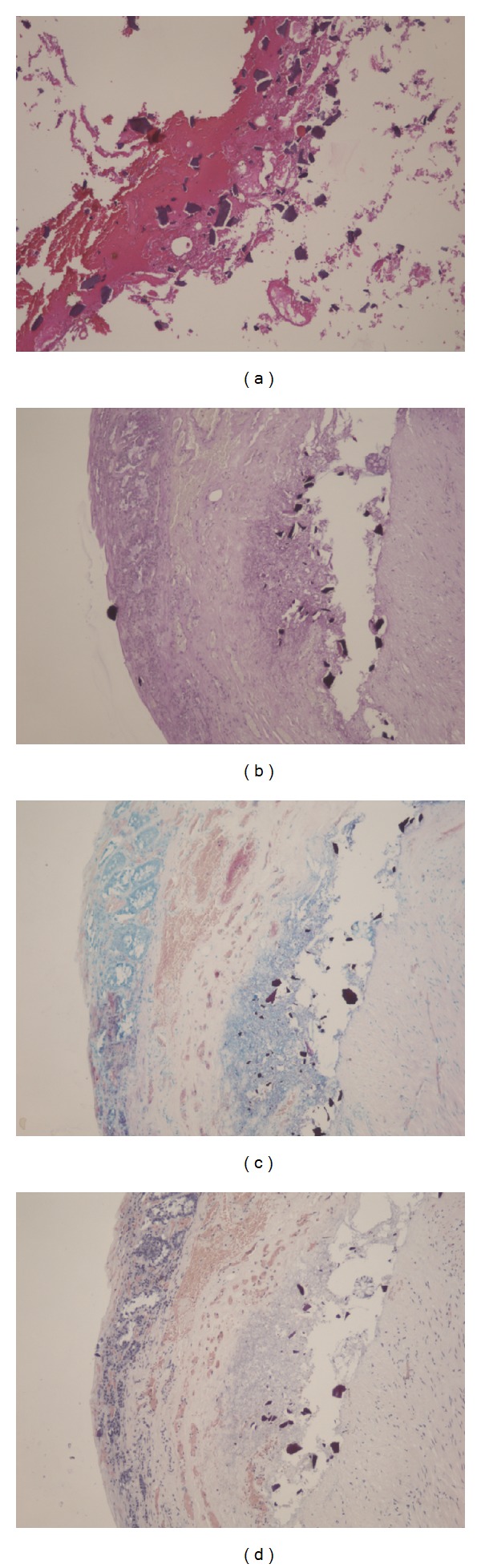
Several basophilic polygonal crystalline materials were found on hematoxylin and eosin staining (a), and crystalline materials were negative on PAS (b), Ziehl-Neelsen (c), and Congo-red stains (d).

A 90-year-old female complaining of severe upper abdominal pain was transferred to our institution in December 2012. The patient's history included angina pectoris, which was treated using coronary stents and dual antiplatelet therapy. The patient was treated with hemodialysis for myeloperoxidase-antineutrophil cytoplasmic antibody (MPO-ANCA) associated glomerulonephritis and had been receiving CPS (ARGAMATE) for the treatment of hyperkalemia. On admission, the patient was confirmed to be in shock with the following signs: blood pressure, 77/55 mm Hg; heart rate, 82 beats/min; body temperature, 36.5°C; oxygen saturation, 92% on room air. On clinical examination, her weight, height, and body mass index were 40.6 kg, 139 cm, and 20.9 kg/m^2^, respectively. Inspection of the palpebral conjunctiva revealed evidence of severe anemia. Chest auscultation revealed no evidence of abnormal heart murmurs and no rales or other lung sounds. The abdomen revealed diffuse tenderness, with signs of peritoneal irritation. Physical examination revealed whole body cyanosis. Blood chemical analyses (Table 1) revealed markedly decreased white blood cell counts (1600/*μ*L), severe anemia (red blood cell counts: 234 × 10^4^/*μ*L; hemoglobin levels, 7.9 g/dL), severely decreased platelet counts (6.0 × 10^4^/*μ*L), increased serum urea nitrogen levels (39.3 mg/dL), markedly increased serum creatinine levels (5.35 mg/dL), severe hypoproteinemia (3.5 g/dL), severe hypoalbuminemia (3.0 g/dL), increased glucose levels (199 mg/dL), increased brain natriuretic peptide levels (299.0 pg/mL), mildly increased C-reactive protein levels (1.0 mg/dL), and coagulation dysfunction (prothrombin time, 18%; fibrin/fibrinogen degradation products, 15.6 *μ*g/mL; D-dimer levels, 3.9 *μ*g/mL). Blood gas analyses revealed mild metabolic acidosis (bicarbonate ion carbonic acid, 19.7 mmol/mL; base excess, −4.6 mmol/L). Chest and abdominal radiography revealed free air under bilateral diaphragms, suggesting intestinal perforation (Figures 1(a) and 1(b)). Abdominal computed tomography (CT) revealed the presence of ascites, free air in the abdominal cavity, and the retention of hard stools in the left-sided colon (Figures 2(a) and 2(b)). The diagnosis of intestinal perforation was immediately confirmed; subsequently, the patient underwent emergency surgical treatment. Surgical findings (Figures 3(a) and 3(b)) revealed a 55 × 22 mm perforated site in the middle of the descending colon surrounded with hard stools. No diverticulum was evident in the colon. The descending colon, including the affected site, was resected, and a colostomy was created in the transverse colon. Histopathology of the perforated colon revealed basophilic polygonal crystalline materials, which suggested the association of CPS with this perforation (Figure 4(a)). Crystalline materials were negative for PAS, Ziehl-Neelsen, and Congo-red stains (Figures 4(b), 4(c), and 4(d)). The postoperative course was eventful: the patient went into septic shock and developed acute respiratory distress as well as multiple organ failure. The patient eventually died of panperitonitis on day 3 of admission.

## 3. Discussion

CPS is a cation-exchange resin used in the treatment of hyperkalemia associated with acute or chronic renal failure. Both ARGAMATE (jelly type) and KALIMATE (powder type) contain CPS. The major adverse effect of CPS is constipation. Several cases treated with CPS require purgatives: CPS has been used with a combination of sorbitol liquid, an osmotic purgative. Some authors [1–3] have reported cases that developed intestinal necrosis induced by combined administration of CPS and the sorbitol liquid. Further, colonic ischemia or perforation associated with the combined anal administration of CPS and the sorbitol liquid occurred in approximately 1% of cases [4]. Thereafter, the combined use of CPS and sorbitol was warned. Although the use of CPS alone is permitted, single use of CPS may also cause intestinal necrosis [5].

In patients with chronic renal failure undergoing hemodynamic dialysis (HD), the presence of chronic intestinal inflammation induced by intestinal ischemia should be considered. The renin-angiotensin system (RAS) activated by hypotension following HD, uremia, or hypovolemia may cause vasospasms that include mesenteric vessels, thereby leading to intestinal necrosis and nonobstructive mesenteric ischemia. Under this condition, vulnerable intestinal mucosa may be more easily perforated by hard stools resulting from CPS. Few authors reported a case of direct invasion of CPS from the inflamed intestinal mucosa into the tissue; the vulnerable intestinal mucosa of patients undergoing HD for renal failure was considered as one of the reasons. However, in this case, histopathology of resected specimens revealed no chronic intestinal inflammation caused by vasculitis, thrombus, or ischemic change. Indeed, chronic intestinal inflammation is not very specific as such, and it is not the only sign of the conditions mentioned above. A direct mechanical effect of hard stools following CPS administration may have triggered the colonic perforation in this elderly patient with several comorbidities including old age, poor vascular supply, chronic renal insufficiency, hard stools, and a possible toxic effect of drugs. In the present case, ARGAMATE intake may have worsened the constipation, and a hard stool may have perforated the descending colon. Moreover, the patient lacked dietary fiber because of strict limitations of potassium and fluid intake, which may have aggravated the constipation as well.

**Figure 5:**
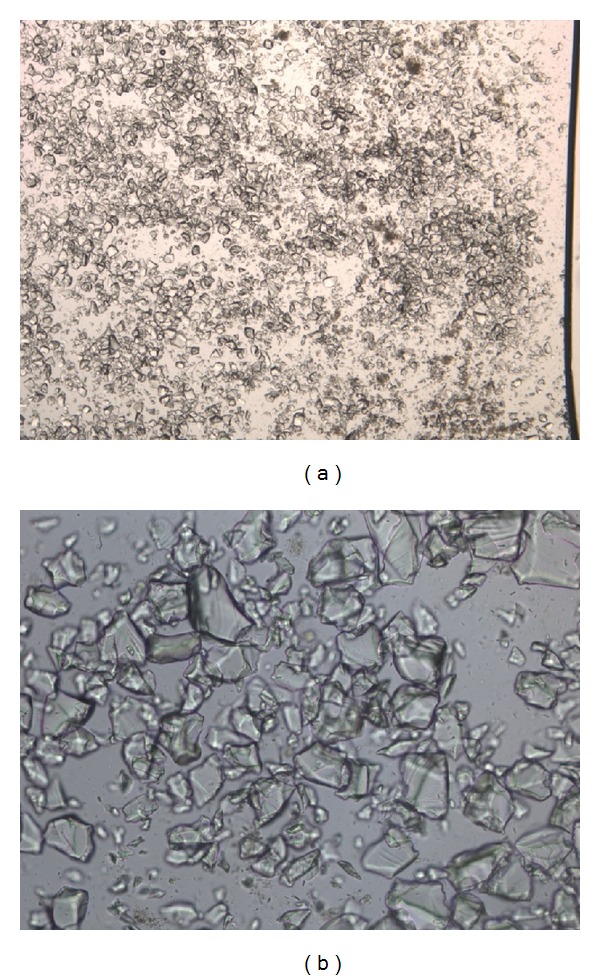
Microscopic examination of ARGAMATE revealed crystalline materials ((a) low power field, (b) high power field).

Microscopic examination of ARGAMATE revealed crystalline fiber materials recognized as CPS (Figures 5(a) and 5(b)). On histopathological analysis, crystalline materials were observed to be scattered around the perforated site following intestinal perforation. The mechanism of this perforation may be similar to that of stercoral colonic perforation. Moreover, crystalline materials were not evident in the nonperforated site of the descending colon suggesting that ARGAMATE directly affected intestines. In most cases, when ARGAMATE causes intestinal perforation, the common site of involvement is the left-sided colon. As intestinal contents move forward to the anus, water is absorbed in intestines and harder stools harden because of the resin; therefore, extremely hard stools are formed in the left-sided colon, including the descending colon, sigmoid colon, and rectum. Moreover, the left-sided colon has several curved portions causing stool obstruction toward the anus. With stagnation of harder stools, colonic pressure increases, leading to colonic perforation or necrosis. Although the possibility remains that ARGAMATE could be the cause of intestinal perforation, there are no reports to date indicating that ARGAMATE causes perforation of the small intestine; therefore, there is no evidence to support this idea.

A previous study reported the [6] use of CPS used in a patient with small intestinal diverticulosis, which led to small intestinal perforation; therefore, the retention and accumulation of CPS in the diverticula may cause direct mucosal damage. In the present case, colonic diverticulosis was not evident in either imaging or surgical findings. Nevertheless, the colonic diverticulum should be observed by imaging or endoscopy before initiating CPS administrations.

## 4. Conclusions

Here we report the case of a 90-year-old female who presented with colonic perforation during CPS administration. When CPS is administered to patients with frequent constipation or the elderly, the risk of intestinal perforation should be considered.

## Figures and Tables

**Table 1:** Laboratory data on admission.

Hematology	
WBC	1,600/*μ*L
RBC	234 × 10^4^/*μ*L
Hb	7.9 g/dL
Ht	23.0%
PLT	6.0 × 10^4^/*μ*L
Coagulation	
PT	68%
PT-INR	3.93
Fib	126 mg/dL
FDP	15.6 *μ*g/mL
D-dimer	3.9 *μ*g/mL
Blood chemistry	
TP	3.5 g/dL
Alb	3.0 g/dL
T-Bil	0.6 mg/dL
*γ*-GTP	7 IU/L
AST	21 IU/L
ALT	11 IU/L
LDH	111 IU/L
BUN	39.3 mg/dL
Cr	5.35 mg/dL
Na	136 mEq/L
K	4.0 mEq/L
Cl	106 mEq/L
BNP	299.0 pg/mL
Sugar	
Glucose	191 mg/dL
Blood gas analysis	
	(Room air)
pH	7.375
PaCO_2_	34.5 mmHg
PaO_2_	72.9 mmHg
HCO_3_^−^	19.7 mmol/L
BE	−4.6 mmol/L
